# Possibilities and Limitations of Prenatal Diagnosis of Rare Imprinting Syndromes: Prader–Willi Syndrome

**DOI:** 10.3390/children13020177

**Published:** 2026-01-28

**Authors:** Simona Anzhel, Nikolinka Yordanova, Emil Kovachev, Darina Krumova, Elis Ismail

**Affiliations:** 1 Department of Obstetrics and Gynecology, Medical University “Prof. Dr. Paraskev Stoyanov”, 9002 Varna, Bulgaria; emil.kovachev@mu-varna.bg (E.K.); elis.ismail@mu-varna.bg (E.I.); 2 Specialized Obstetrics and Gynecology Hospital for Active Treatment “Prof. Dr. D. Stamatov”, 9002 Varna, Bulgaria; 3 Department of Pediatrics, Medical University “Prof. Dr. Paraskev Stoyanov”, 9002 Varna, Bulgaria; nikolinka.yordanova@mu-varna.bg (N.Y.); darina.krumova@mu-varna.bg (D.K.); 4 Department of Pediatrics, University Hospital “St. Marina”, 9002 Varna, Bulgaria

**Keywords:** Prader–Willi syndrome, prenatal diagnosis, intrauterine growth restriction, reduced fetal movements, imprinting disorders, methylation analysis

## Abstract

**Background:** Prader–Willi syndrome (PWS) is a multisystemic complex imprinting disorder. Prenatal diagnosis of PWS is still a challenge with non-specific ultrasound markers and limitations for diagnosis with non-invasive screening methods. Prenatal suspicion and early postnatal diagnosis are essential for promoting healthy growth and development, preventing complications, and providing healthcare professionals and families with the necessary support and resources for effective management. **Presentation:** We report two PWS cases caused by maternal uniparental disomy, who presented with IUGR, characterized by reduced fetal abdominal circumference (AC) in the second and early third trimesters, reduced fetal movements, normal Doppler indices and oligohydramnios. They were diagnosed in the early neonatal period with no prenatal suspicion but with similar ultrasound markers of the developing pregnancies, analyzed retrospectively. **Aim:** This study aims to emphasize the need to raise awareness among specialists about genetic syndromes such as Prader–Willi syndrome, to improve the information provided to couples regarding the limitations of current prenatal screening methods, as well as to ensure that, in cases of prenatal suspicion, appropriate genetic testing can be initiated. A confirmed diagnosis would allow timely and adequate measures to be taken, given the complications of the postnatal period in these patients and their need for specialized care and management. **Conclusions:** The presence of the aforementioned prenatal characteristics may raise suspicion for PWS. In such cases, invasive diagnostic procedures and methylation testing may be indicated, enabling earlier diagnosis and timely management, which can ultimately improve the quality of life of affected individuals and their families.

## 1. Introduction

PWS was first described by Langdon Down in 1887; however, it was not formally described in the medical literature until 1956, when Prader, Labhart, and Willi reported nine cases (five boys and four girls, aged 5–23) with similar clinical symptoms [1,2]. It is now recognized as a genetic imprinting disorder with a birth incidence of 1/15,000–25,000 worldwide (3.1 per 100,000 live births in Europe) and affecting males and females equally. There are three main genetic subtypes in PWS: paternal 15q11–q13 deletion (65–75% of cases), maternal uniparental disomy 15 (20–30% of cases), and imprinting defect (1–3%) [3]. DNA methylation analysis is the only technique that can diagnose PWS across all three molecular genetic classes and differentiate PWS from Angelman syndrome. Unfortunately, prenatal detection of PWS remains limited due to non-specific ultrasound findings and inability of noninvasive screening to reliably guide invasive diagnostic testing with methylation analysis. There is a need to raise awareness among healthcare providers regarding the potential clinical signs that may lead to suspicion of underlying genetic condition in certain pregnancy complications during the late second and third trimester [2,4,5]. Noninvasive prenatal testing (NIPT) can detect the deletion subtype of Prader–Willi syndrome but cannot identify cases caused by maternal uniparental disomy or imprinting defects, which together account for up to one third of patients. Therefore, a normal NIPT result does not exclude PWS, and DNA methylation analysis remains the only method with near-100% sensitivity for all molecular classes.

Analyses have identified significant discrepancies between Prader–Willi syndrome caused by paternal deletions and those caused by maternal uniparental disomy 15 (UPD15). Importantly, UPD-related PWS is characterized by milder and less recognizable prenatal and neonatal phenotypes, frequently lacking major structural anomalies and often escaping detection by noninvasive prenatal testing. As a result, pregnancies affected by UPD-PWS are at particularly high risk of being falsely reassured despite the presence of subtle functional fetal abnormalities. The frequency of chromosome 15 nondisjunction may increase exponentially with maternal age. Children born to older mothers have a higher rate of maternal uniparental disomy of chromosome 15 compared with those born to mothers younger than 35 years [6,7].

PWS is difficult to diagnose prenatally due to the lack of accurate, well-characterized fetal phenotypes that would otherwise provide a basis for subsequent molecular genetic studies. To date, only a few reports of the diagnosis of PWS during pregnancy have been published [3,4,5,8]. Most of them describe non-specific prenatal signs suggestive of PWS. They are incidentally identified as trisomy 15 during invasive diagnosis by chorionic villus sampling or amniocentesis for other established abnormalities during pregnancy. Prenatal diagnosis of paternal chromosome 15 translocation is more commonly reported [8]. In addition, increased nuchal translucency and abnormal serum maternal screening in the first trimester may indicate the need for invasive testing and can lead to an incidental diagnosis of PWS [3].

Reported prenatal findings associated with PWS are non-specific and usually have a late onset in the second half of pregnancy which include abnormal position of feet and toes [4], polyhydramnios [4,8,9], decreased fetal activity [4,10], hypoplasia of external genitalia [11], breech position [4], diminished fetal movement [4,8], intrauterine growth restriction (IUGR) [8,12], facial dysmorphism [4], and increased head to abdominal circumference and decreased abdominal circumference [4].

We present two cases of postnatal diagnosis of PWS in newborns with normal noninvasive prenatal testing results, including exclusion of PWS and normal fetal morphology scans. Retrospective analyses of the ultrasound protocols raised suspicion of nonclassical and non-characteristic signs in both pregnancies, described in the late third trimester.

The purpose is to increase awareness among obstetricians and gynecologists that rare imprinting syndromes like PWS may not be reliably detected or excluded by current prenatal testing and fetal morphology scans, and parents should be appropriately counseled. For this reason, pregnancies complicated by UPD-related PWS represent an ideal model to study the limits of current prenatal screening strategies and the potential value of phenotype-driven invasive testing.

The novelty of this report lies in the demonstration that maternal UPD-related Prader–Willi syndrome is associated with a reproducible pattern of late-onset functional fetal abnormalities—reduced abdominal circumference growth, reduced fetal movements, abnormal amniotic fluid volume and normal Doppler indices—which can be retrospectively recognized and prospectively used to guide targeted prenatal genetic testing. This approach moves beyond isolated ultrasound markers and provides a clinically actionable prenatal pattern for a genetic subtype that is usually missed by standard screening.

## 2. Case Presentation

### 2.1. Case 1

A 43-year-old primiparous woman was admitted for an elective cesarean section (CS) following a high-risk pregnancy. This was her fourth pregnancy achieved through in vitro fertilization (IVF), with a history of three previous pregnancy losses: one spontaneous abortion, one missed abortion, and one termination due to fetal diagnosis of Down syndrome.

Her obstetric history was notable for two prior myomectomies and four hysteroscopic procedures. The current pregnancy progressed uneventfully. Routine fetal morphology assessments and basic noninvasive prenatal testing (NIPT) showed no abnormalities. Non-stress test (NST) maintained normal variability and reactivity throughout, and amniotic fluid volume remained within physiological limits.

Retrospective analysis of the ultrasound data revealed a pattern of abnormal fetal behavior and growth disproportion, including persistent difficulty in visualization of the fetal face and genitalia due to reduced and restricted fetal movements, as well as subtle asymmetric growth with relatively preserved head circumference compared to abdominal circumference.

**Figure 1:**
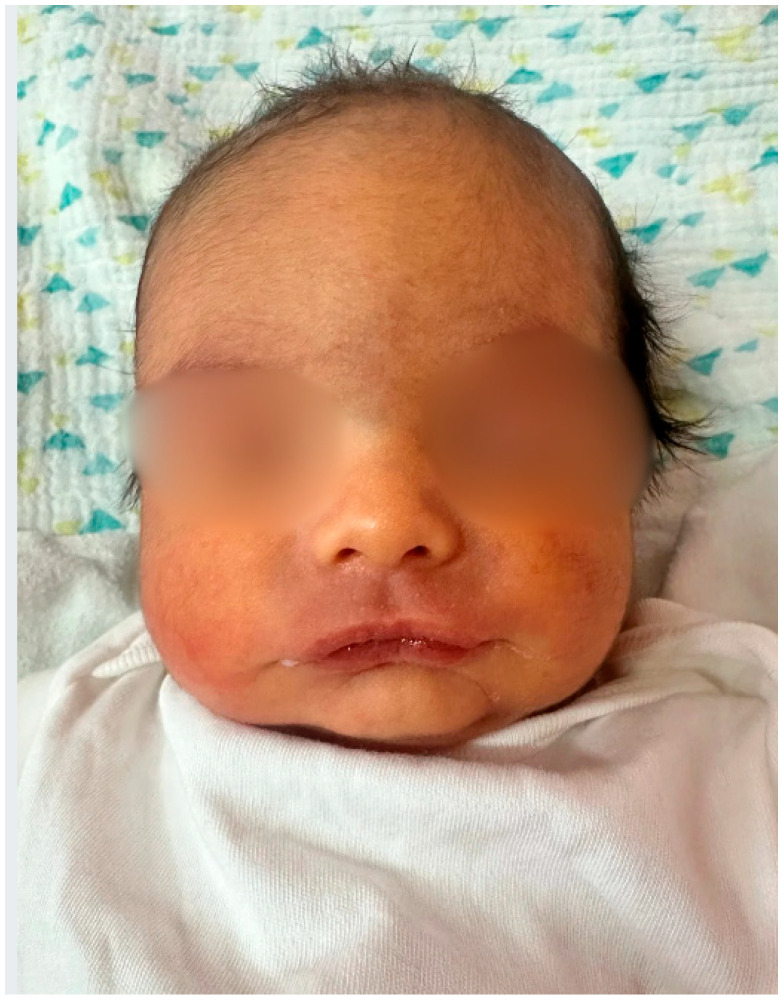
Frontal facial view of the male neonate (Case 1) on the second day of life, demonstrating increased interorbital distance, narrow bifrontal diameter, almond-shaped eyes and mild facial dysmorphism, features characteristic of Prader–Willi syndrome.

**Figure 2:**
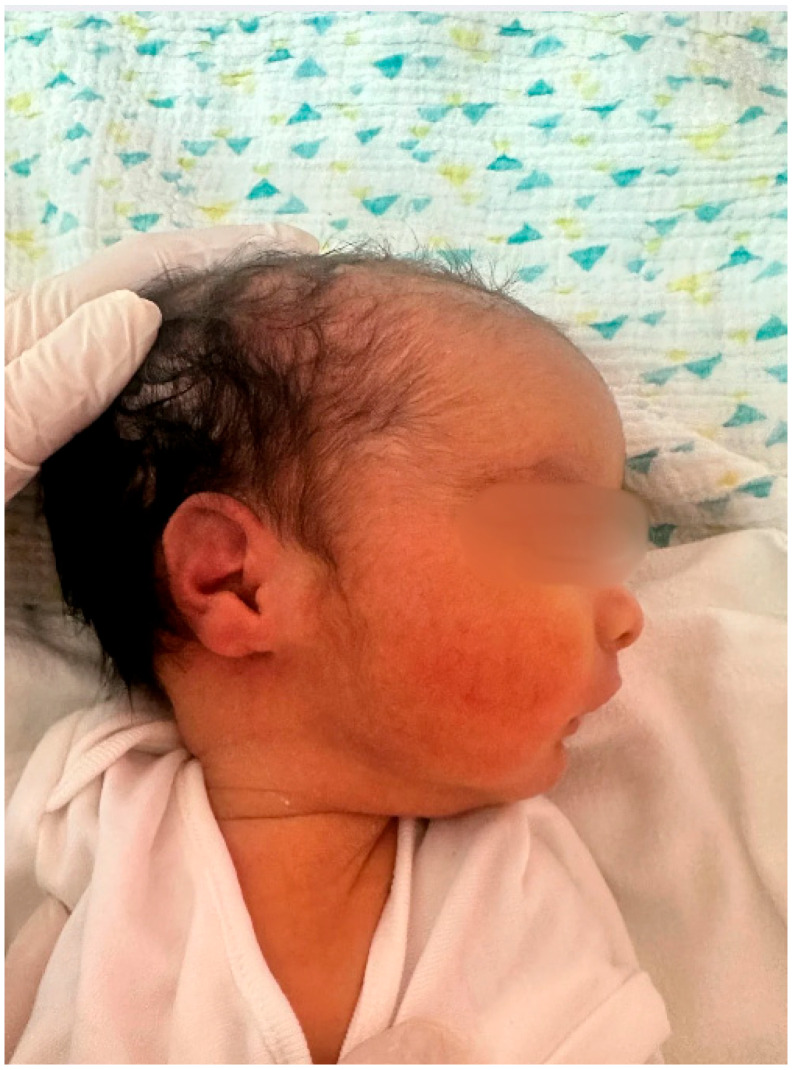
Lateral facial profile of the male neonate (Case 1) showing retrognathia and recessed chin, reflecting abnormal craniofacial development commonly associated with Prader–Willi syndrome.

**Figure 3:**
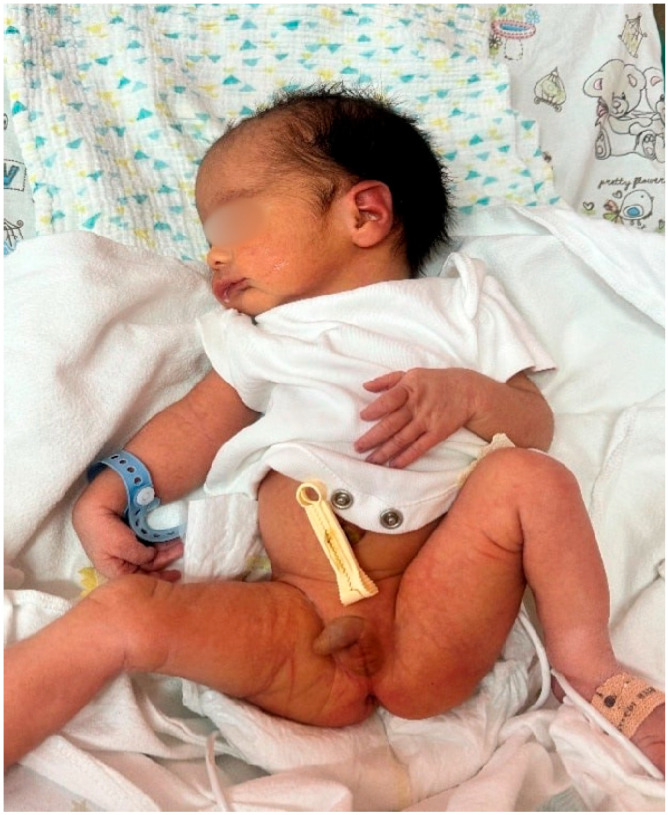
Neonatal examination of Case 1 demonstrating generalized muscular hypotonia and bilateral cryptorchidism, which are hallmark manifestations of hypothalamic–pituitary dysfunction in Prader–Willi syndrome.

At 35 weeks of gestation, due to the onset of genital bleeding and signs of active labor, the patient underwent cesarean delivery. A live male infant was delivered, with a birth weight of 2810 g and a birth length of 48 cm. Postnatal examination revealed bilateral cryptorchidism and subtle craniofacial dysmorphism (Figure 1 and Figure 2), followed by progressive generalized hypotonia and feeding difficulties on the second day of life (Figure 3), raising suspicion of an underlying genetic syndrome.

On the second day of life, the neonate exhibited progressive muscular hypotonia and feeding difficulties (Figure 3). A pediatric consultation raised clinical suspicion for PWS. The infant was subsequently referred for comprehensive medical-genetic evaluation and counseling, and the diagnosis was confirmed for maternal uniparental disomy.

### 2.2. Case 2

A multiparous woman was admitted for cesarean section at 36 weeks of gestation. Indications for early operative delivery included a history of previous cesarean section, oligohydramnios, and intrauterine growth restriction (IUGR) noted since the 20th week of gestation with predominant reduction in the fetal abdominal circumference and normal umbilical and middle cerebral artery Doppler values, making placental insufficiency unlikely and suggesting a possible genetic etiology. Additionally, non-stress test (NST) revealed reduced to absent variability and reactivity, despite normal Doppler flow measurements.

**Figure 4:**
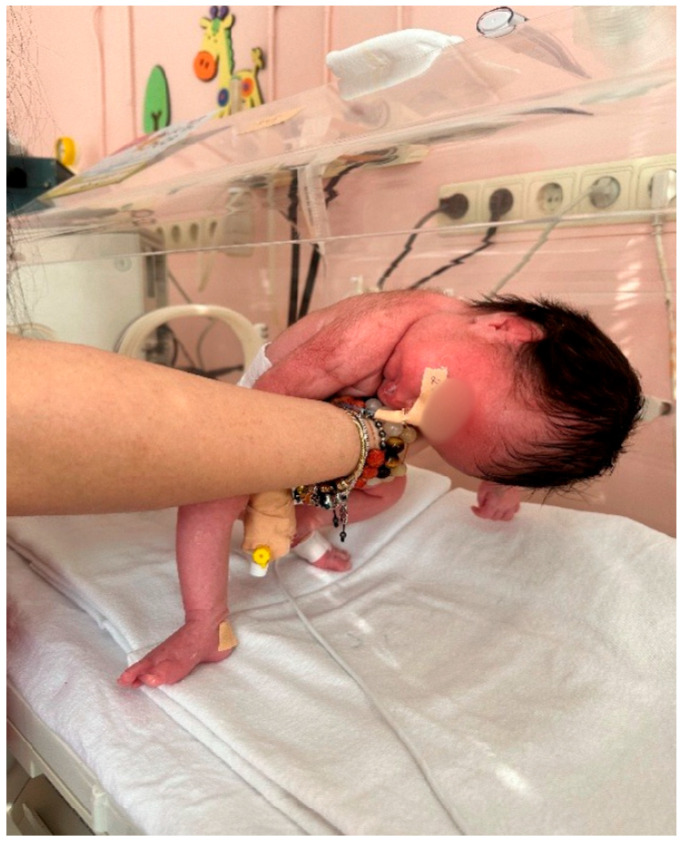
Neonate (Case 2) with severe generalized hypotonia, illustrating the impaired neuromuscular tone typical for Prader–Willi syndrome in the early neonatal period.

**Figure 5:**
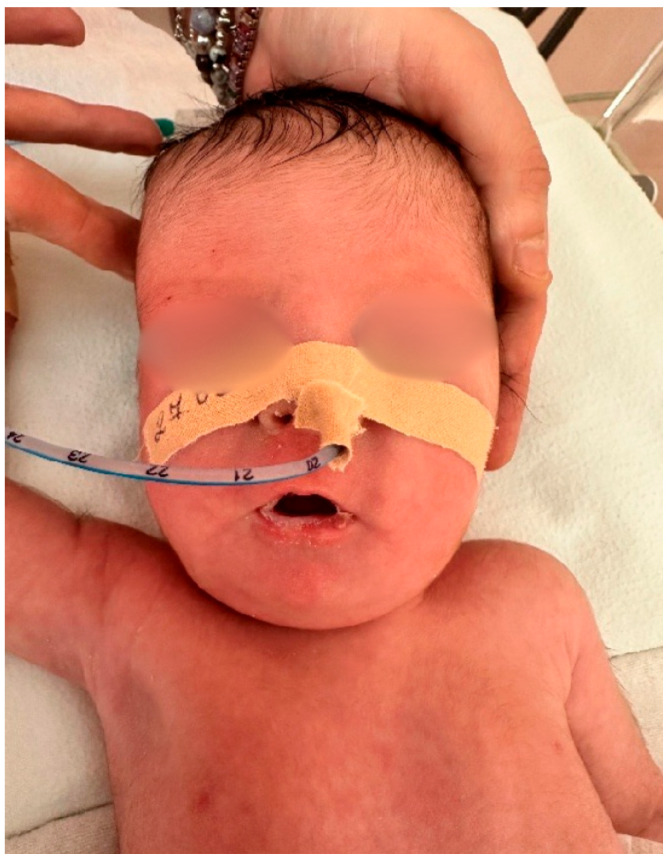
Facial phenotype of the neonate (Case 2) showing narrow bifrontal diameter, almond-shaped eyes, short nose, thin upper lip and downturned corners of the mouth, representing the characteristic dysmorphic pattern of Prader–Willi syndrome.

Following delivery, the newborn (birth weight 2225 g, birth length 46 cm) exhibited signs of respiratory compromise, including acrocyanosis, tachydyspnea, increased oxygen requirements, and feeding difficulties. Neuromuscular assessment revealed progressive muscular hypotonia (Figure 4), together with typical Prader–Willi facial dysmorphism, including almond-shaped eyes, narrow bifrontal diameter, and downturned mouth corners (Figure 5).

A working diagnosis of PWS was established and subsequently confirmed by DNA methylation analysis, which demonstrated maternal uniparental disomy of chromosome 15, consistent with the genetic subtype identified in Case 1.

In light of the clinical presentation and phenotypic characteristics, both neonates were referred to the Expert Center for Rare Endocrine Diseases at “Sveta Marina” University Hospital, Varna, Bulgaria, for further diagnostic evaluation and treatment. A confirmed diagnosis of PWS allows for timely and adequate measures to be taken, given the complications of the postnatal period in these patients and their need for specialized care and management. The key prenatal ultrasound findings for both cases are summarized in Table 1. Exact biometric percentiles and Z-scores were not consistently available from the retrospective ultrasound records; therefore, growth patterns are reported as relative and disproportionate changes, which are clinically more relevant for syndromic suspicion than isolated absolute values.

Genetic confirmation of Prader–Willi syndrome in both patients was performed using DNA methylation-specific analysis of the 15q11–q13 region. In both cases, abnormal maternal-only methylation patterns were detected, consistent with maternal uniparental disomy of chromosome 15. Additional karyotyping and microarray analysis excluded large deletions or duplications.

## 3. Discussion

Both presented cases showed a remarkably similar prenatal–postnatal pattern, consistent with previously described phenotypes of Prader–Willi syndrome due to maternal uniparental disomy 15. These similarities in prenatal ultrasound findings are summarized in Table 1. Unlike deletion-type PWS, UPD cases often lack structural anomalies and abnormal NIPT results but display functional fetal abnormalities such as reduced movements, growth disproportion, and abnormal amniotic fluid volume. The overlapping features in our two patients support the existence of a recognizable, although subtle, prenatal phenotype for UPD-related PWS.

The postnatal phenotypes illustrated in Figure 1, Figure 2, Figure 3, Figure 4 and Figure 5 demonstrate the classic craniofacial and neuromuscular manifestations of Prader–Willi syndrome, supporting the genetic diagnoses and linking the subtle prenatal findings with the fully expressed neonatal phenotype.

Although PWS occurs in approximately 1 in 15,000 to 1 in 30,000 live births, the syndrome is underdiagnosed or misdiagnosed in the neonatal period due to its non-specific early manifestations [13]. This highlights the critical need for heightened clinical suspicion among obstetricians, neonatologists, and pediatricians, particularly in the presence of suggestive features such as severe hypotonia, feeding difficulties, poor weight gain despite low birth weight, and facial dysmorphisms.

The most common ultrasound feature noted in published reports is the presence of fetal growth retardation (usually abdominal circumference below the 5th percentile) and polyhydramnios, which in some cases may be severe enough to require amnioreduction [14]. This is likely due to an impaired swallowing reflex resulting from the associated skeletal muscle hypotonia. Although polyhydramnios is more commonly reported in fetuses with Prader–Willi syndrome due to impaired swallowing, oligohydramnios may also occur, particularly in late gestation. Reduced fetal movements and hypotonia may lead to decreased fetal breathing and swallowing activity, thereby altering the balance of amniotic fluid regulation. In addition, growth-restricted fetuses with neurodevelopmental impairment may exhibit reduced urine output and altered placental–fetal fluid exchange, contributing to oligohydramnios. The presence of oligohydramnios in Case 2, therefore, further supports a functional fetal disorder rather than placental insufficiency. The phenotype of PWS includes persistent severe hypotonia, facial dysmorphism with a narrow bifrontal head diameter, almond-shaped eyes, short nose, downturned corners of the mouth, thin upper lip, smoothed philtrum, lighter skin tone than the parents, acromicry with elongated and pointed fingers, hypogonadism with micropenis, hypoplastic scrotum, and cryptorchidism in boys, and hypoplastic labia majora in girls [15].

Reduced fetal movements are reported in 4–16% of pregnancies with euploid fetuses, in most cases, transient and clinically insignificant [4,15]. However, in some cases, this can be a sign of congenital musculoskeletal defects or fetal distress.

Intrauterine growth restriction (IUGR) is most often due to placental insufficiency, especially with an increased AC/HC ratio and abnormal umbilical artery Doppler values. Normal Doppler values exclude placental insufficiency, and IUGR may be an indirect sign of underlying genetic disease [5].

Cryptorchidism is present in 4% of healthy full-term male infants and 1–2% of healthy boys during the first year of life. It may be an isolated minor anomaly or part of various genetic and endocrine syndromes. Bilateral cryptorchidism has been reported to occur in almost all infants with PWS [15]. Screening for cryptorchidism is recommended in the presence of other suspicious findings for fetal PWS [16].

A study of 106 families, 47 of whom had a child with PWS aged up to 10 years, was conducted. When analyzing data from ultrasound examinations during pregnancy and history taken from mothers, the authors found that reduced fetal movements, small for gestational age fetuses (SGA), asymmetric retardation (increased HC/AC ratio), and polyhydramnios were found in 88%, 65%, 43% and 34%, respectively. No concomitant morphological defects were found. The authors recommend that prenatal genetic screening for PWS by methylation testing may be recommended in the combination of polyhydramnios, SGA, or asymmetric growth retardation with normal Doppler findings [5].

The early recognition of PWS has substantial implications for patient outcomes. In the early neonatal period, impaired respiratory control in PWS is believed to arise from a combination of hypothalamic dysfunction, immature brainstem respiratory circuits, blunted chemosensitivity, and generalized hypotonia. Loss of paternally expressed genes in the 15q11–q13 region—whether due to paternal deletion or UPD—disrupts the development and modulation of central respiratory networks, leading to reduced ventilatory responses to both hypercapnia and hypoxia [17,18]. Clinically, neonates with PWS may present with central apnoeas, hypoventilation, and oxygen desaturations [19,20]. Several reports describe newborns requiring supplemental oxygen due to persistent hypoxaemia or central apnoeas [21,22]. Case series show that low-flow oxygen can reduce central apnoeic events and improve oxygenation without worsening hypoventilation in infants with PWS, suggesting that oxygen therapy may serve as a supportive intervention until respiratory control matures, which typically improves over the first months of life [22,23]. Together, this evidence indicates that neonatal respiratory instability in PWS reflects a primary disturbance of central ventilatory regulation with impaired chemoreflexes, for which careful monitoring—and, in selected neonates, supplemental oxygen—may be beneficial. This approach was also applied in our two patients after their referral to an Expert Center (initially in the intensive care unit, and subsequently in the regular ward).

Other supportive care needed in the neonatal period includes targeted nutritional management and early physiotherapy. In many neonates with Prader–Willi syndrome (PWS), severe hypotonia and poor suck–swallow coordination often requires temporary nasogastric tube feeding to ensure adequate caloric intake and avoid prolonged failure to thrive [24]. Early, closely supervised tube feeding also supports a smoother transition to oral feeding as oromotor function improves [25]. Given the profound axial hypotonia and delayed motor milestones typical of PWS, early physiotherapy focusing on postural control and antigravity activation is strongly recommended [26]. Structured early motor-training programs have been shown to advance the acquisition of sitting and walking, particularly when combined with growth hormone therapy [27]. Early initiation of recombinant human growth hormone (rhGH) therapy, after appropriate clinical assessment, is increasingly recognized as a key component of comprehensive early care. Studies in infants and toddlers demonstrate that rhGH started within the first years of life improves linear growth and body composition and accelerates motor development [25,28]. In the two patients presented, all necessary measures were implemented in a timely manner, resulting in favorable outcomes for their physical and neuropsychological development. This further emphasizes the importance of raising awareness about rare imprinting syndromes already at the stage of early prenatal diagnostics and allows for timely referral to multidisciplinary care teams and genetic counseling services, which are essential for the early-intervention model and long-term management.

## 4. Conclusions

These cases illustrate that pregnancies affected by maternal uniparental disomy-related Prader–Willi syndrome may present prenatally with a constellation of subtle but consistent functional abnormalities, rather than with structural malformations. When intrauterine growth restriction is driven mainly by reduced abdominal growth and accompanied by decreased fetal activity and normal placental Doppler findings, this pattern should be interpreted as suggestive of a non-placental, genetic etiology.

Clinical message: When obstetricians encounter a fetus with unexplained growth disproportion and reduced movements in the presence of normal Doppler indices and normal NIPT, Prader–Willi syndrome—particularly the maternal UPD subtype—should be actively considered, and invasive testing with methylation analysis should be discussed with the parents.

## Figures and Tables

**Table 1:** Comparison of prenatal ultrasound and monitoring findings in the two cases.

Parameter	Case 1	Case 2
Gestational age at delivery	35 weeks	36 weeks
Onset of growth restriction	Late second/early third trimester	From 20th week
Abdominal circumference (AC)	Disproportionately reduced	Disproportionately reduced
Head circumference (HC)	Relatively preserved	Relatively preserved
HC/AC ratio	Increased	Increased
Fetal movements	Reduced	Markedly reduced
Amniotic fluid volume	Normal	Oligohydramnios
Umbilical artery Doppler	Normal	Normal
Middle cerebral artery Doppler	Normal	Normal
Non-stress test (NST)	Normal reactivity	Reduced to absent reactivity
Structural anomalies on ultrasound	None detected	None detected
Noninvasive prenatal testing (NIPT)	Normal	Normal

## Data Availability

The data presented in this study are available on request from the corresponding author. The data are not publicly available due to privacy and ethical reasons.

## Abbreviations

AC	abdominal circumference
HC	head circumference
IUGR	intrauterine growth restriction
NIPT	noninvasive prenatal testing
NST	non-stress test
PWS	Prader–Willi syndrome
rhGH	recombinant human growth hormone
SGA	small for gestational age
UPD	uniparental disomy
